# Duration, score, and timing: factors influencing the success of offensive transitions in top, marginal, and emerging football leagues

**DOI:** 10.3389/fspor.2024.1462932

**Published:** 2025-01-10

**Authors:** Pedro Eusebio, Pablo Prieto-González, Rui Marcelino

**Affiliations:** ^1^ University of Maia, Maia, Portugal; ^2^ Research Centre in Sports Sciences, Health Sciences and Human Development, CIDESD, CreativeLab Research Community, Vila Real, Portugal; ^3^ Sport Sciences and Diagnostics Research Group, College of Humanities and Sciences, Prince Sultan University, Riyadh, Saudi Arabia; ^4^ Football School, Portuguese Football Federation, Oeiras, Portugal

**Keywords:** performance analysis, counterattacks, offensive transition, positive outcome, time, soccer, game moments

## Abstract

**Objective:**

The study aimed to increase our understanding of offensive transitions in football, focusing on the time spent executing them and how it is influenced by the period in which they occur and the instant score. The objective is to understand its temporal variables and how they influence the unfolding of the scoreline during a match.

**Methodology:**

This study analyzes 1,649 goals resulting from offensive transitions in 1,151 games across three leagues categories: Top, Marginal, and Emerging leagues. Multinomial logistic regression was performed to understand associations and predictors between the variables: “duration of a transition”, “Time of offensive transition occur” and the “score at the moment”.

**Results:**

The research reveals that, across all league categories, the last 15 min of the first and second halves, have the highest frequency of successful offensive transitions, particularly on the last 15 min of matches. In Top leagues, 69% of offensive transitions last for 9 s or more, with slower transitions more prevalent towards the end of games. In Marginal leagues, 41% of successful transitions took 13 s or longer, while in Emerging leagues, 53% of successful offensive transitions occurred when the game was not in a 0–0 state. The results show associations between all variables in Top Leagues. No associations were found in Marginal leagues. Associations between duration of the transition and the time of the game they occur were found on Emerging Leagues.

**Conclusion:**

These findings underscore the importance of tailored strategic plans for offensive transitions, to optimize scoring opportunities and performances.

## Introduction

In the captivating realm of football, where every pass, every sprint, and every strategic decision carries the weight of victory or defeat, the intricacies of offensive transitions have emerged as a pivotal determinant of a team's ultimate success (1). Exploring these transitions across different leagues provides valuable insights into how context—both tactical and cultural—shapes these pivotal moments. Football competitions around the world are influenced by distinct cultural, developmental, and tactical factors. European leagues, such as those in Spain, Italy, Germany, Portugal, the Netherlands, and Russia, are known for their emphasis on tactical sophistication, strategic organization, and fast-paced transitions. In contrast, leagues in the Middle East, such as those in Qatar, Saudi Arabia, and the UAE, often focus more on technical skills and may exhibit different tactical priorities, influenced by the unique footballing culture and resources available in these regions (2). These contrasting environments in European and Middle Eastern leagues provide a deeper understanding of how offensive strategies vary across continents (3). As the game continues to evolve, it is imperative to dissect and comprehend the multifaceted role that offensive transitions play in shaping the final score (4).

Offensive transitions refer to the phase when a team regains possession and shifts from defense to attack, often exploiting moments of disorganization in the opponent's setup. Some transitions lead to immediate goal-scoring chances, while others require a more patient buildup to maintain possession. Within this framework, counter-attacks represent a distinct, faster-paced subset of offensive transitions, marked by rapid forward play designed to take advantage of an opponent's scattered defense (4, 5).

While time remains an omnipresent factor, this study delves into a novel perspective, wherein the speed of offensive transitions is not decisive, but relevant and critical to obtain success.

In 2011, Casal et al, stated that offensive transitions when started by a direct recovery of the ball last for an average of 16 s. Although empirically it is perceived that quick offensive transitions can take advantage of the opposing team's disposition (6), strategic decision-making remains critical for successful results to occur. Several studies have shown that fast attacks resulted in a higher conversion rate of shots per goal than slower attacks (7, 8). Another factor that can influence the time the offensive transition takes is the proximity of the ball's recovery to the opponent's goal. According to Larson (9) the likelihood of a successful offensive transition increases depending on the proximity of the ball recovery that is, recoveries made in areas close to the opposing goal have a greater probability of success. Thus, the loss of possession zone, which is the same as the zone from which the offensive transition begins, has been highlighted by different authors since it influences their effectiveness, transition time and success (10, 11). However, Turner & Sayers (9, 12), in their study on transition moments in the A-League competition, highlighted the need for further investigation on how transition speed is affected by other variables and what role speed plays in successful decision-making. The same authors (12) further note that positive and negative outcomes can occur irrespective of transition speed, and that no differences were found between the mean transition speeds of positive and non-positive outcomes. The available data shows that while the swiftness of the gameplay is undeniably essential, it does not determine the success of these crucial moments.

The possibility of this paradigm shift challenges and put in perspective the prevailing notion that speed alone is paramount. The astute and calculated strategic decision-making is the major determinant in the offensive transitions. When the players flag the opportunity of an offensive transition, it is the shrewd selection of passes and movements, as well as the keen assessment of spatial, and judicious timing that paint the outcome of that moment of play (13). It will also have to be considered that winning teams tend to repeat behavior patterns throughout the games, including formations and styles of play (14). This suggests that to score goals, successful teams tend to replicate certain variables associated with their success, namely the decision-making at specific moments of the game. In this sense, the teams that manage the data proficiently and take from their a good analysis will obtain competitive advantages (13, 15), since the tactical behaviors of the opponents and their team are anticipated (13, 16).

This study aimed to determine the time spent in the execution of a successful transition in different league groups. The study also aimed to provide information about the playing time (interval) in which offensive transitions occur, how this relates to the time spent executing them, and how it is influenced by the “score at the moment”. The ultimate aim was to increase our understanding of offensive transitions in football and their contribution to goal scoring, thus contributing to advancing tactical knowledge in the sport.

## Materials and methods

### Sample and variables

This study can be classified as observational Multidimensional Nomothetic (17). This investigation utilized a sample of 1,151 games from the 2019–20 season, encompassing the games played in the Emerging, Marginal, and Top Leagues analyzed, in all nine leagues from the season's commencement until the midpoint of the season, leading to 3,497 goals. The leagues were grouped into three groups of three leagues. The Top Leagues group is comprised of the Spanish, Italian and German leagues, then classified as 2nd, 3rd, and 4th in the UEFA ranking (at the date the events were collected) and which are the leagues with the highest turnover and value invested in the transfer markets. The Marginal leagues, consisting of the Portuguese, Dutch, and Russian leagues, which are in 6th, 7th, and 8th places, respectively, and which present a high number of transfers from these to the others analyzed leagues, and the Emerging Leagues, (EL), made up of the Qatar, Saudi Arabia, and UAE leagues that appear as markets with high financial potential that manage to attract some international players but those leagues are played mainly by local players.

**Figure 1:**
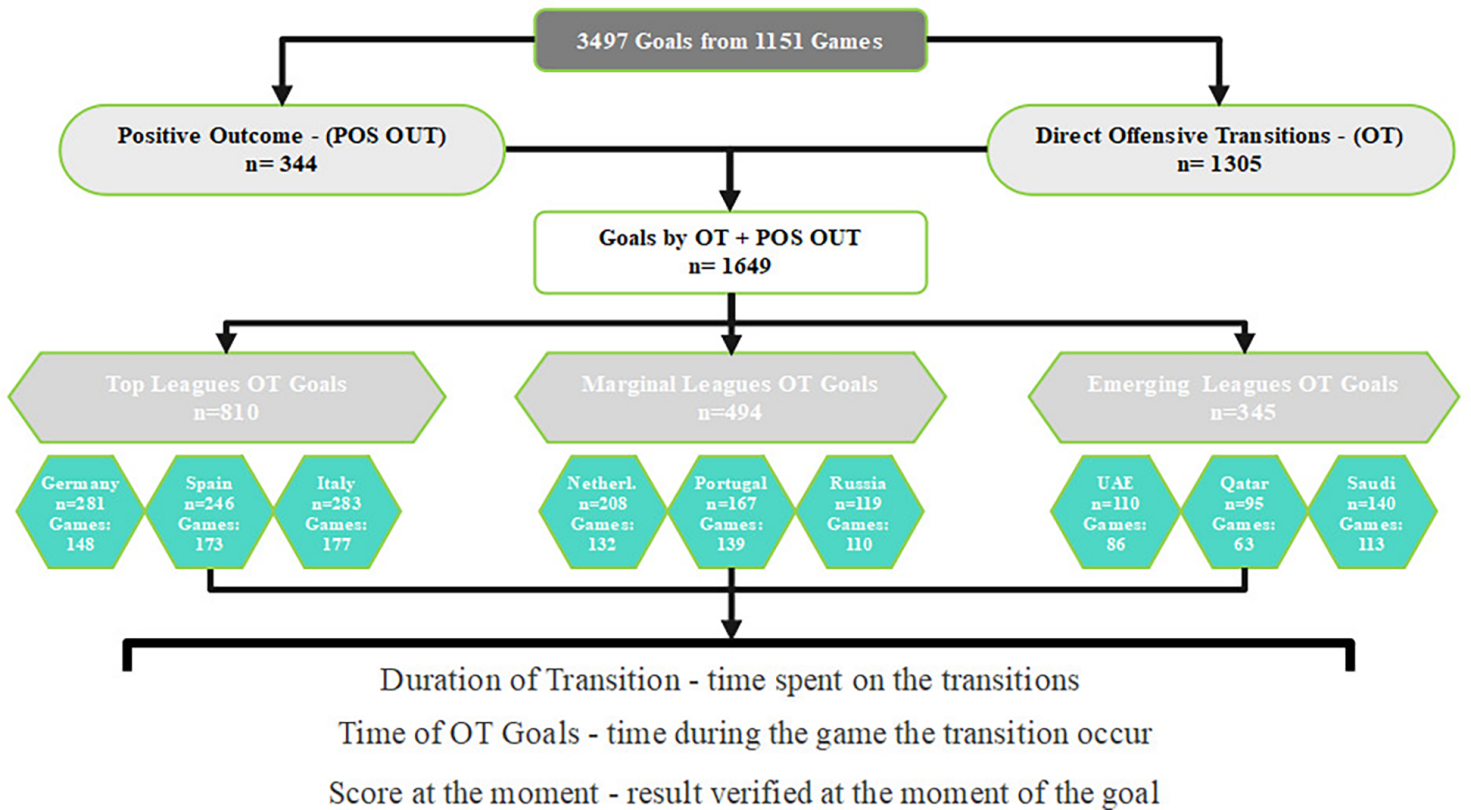
Questions algorithm. All goals were subject to analysis. For this investigation, only goals from Offensive transitions and Positive Outcome were considered, represented by OT Goals.

All goals were categorized into three types: (i) Non-offensive Transition (NT)—goals scored without a preceding offensive transition, or a set pieces, i.e., goals that arise from stablished attacks, (ii) Direct Offensive Transition (OT)—goals that result directly from a change in possession, where a team regains the ball and immediately attempts to score, and (iii) Set Pieces (SP)—Goals scored from set pieces. The set pieces were analyzed based on the precedent play. If the play before the fault was not a transition, the goal stayed as Set Pieces, however, if the play was an offensive transition play, the goal was classified as a Positive Outcome (POS OUT). A total of 1,305 goals were obtained through direct offensive transitions, with 344 stemming from positive outcomes, resulting in 1,649 goals from offensive transitions. The goals classified as Positive Outcome (POS OUT) were treated as Offensive Transition (OT), with the end of the play being considered the moment of the positive outcome. Therefore, these goals were only attributed to offensive transition without differentiation (see Figure 1).

### Procedures

The instrument used for the analysis was adapted from the system proposed by Turner and Sayers (12), along with an observation and registration instrument proposed by the authors, constituted by systems of categories, that precisely define the target registration criteria. The videos were obtained from two video providers, InStat and Wyscout. To maintain methodological rigor and ensure comparability across teams, only games with goals from the first round to the midway point of each championship were included in the analysis.

As a result, all teams were subject to an equivalent number of observations, and every team in each championship played with each other. All plays resulting in a goal were analyzed from the moment the ball was recovered until the goal was scored. All plays that resulted in a goal were analyzed from the moment the ball was recovered until the goal was scored. This includes scenarios where a team gains or regains ball control through a tackle, interception, or rebound. In cases of quick restart of the game that led to an offensive transition, the moment of the beginning of the first action, such as a throw-in, goal kick, or free kick, was considered. The goals scored by Offensive Transition (OT) were meticulously described, focusing on the following variables: (i) the time during the game the transition occurs; (ii) the time spent on the transition; (iii) the “score at the moment” of the transition.

The variable “duration of Transition” takes into account the time elapsed between the beginning and end of the transition, categorizing it into three categories: Fast (0″ to 8″), Medium (9″ to 12″), and Slow (13″ and above). “Time of OT occur” considers the elapsed game time when the transition occurs, with intervals defined in 15 min increments: 0′ to 15′, 16′ to 30′, 31′ to 45′ to 60′, 61′ to 75′, and 76′ to 90′**.

Lastly, the variable “Score at the moment” accounts for the scoreline at the moment the transition leads to a goal, distinguishing between three scenarios: “Tied” when the goal is obtained with the score at 0–0, “Balanced” when the goal is obtained with a score difference of one goal, and “Unbalanced” when the goal is obtained with a score difference of two or more goals. Table 1 it helps to understand the algorithm of the different variable's options.

**Table 1:** Variables and respective categories analyzed.

Variable	Category
Duration of Transition	Fast: 0″ to 8″; Medium: 9″’ to 12″; Slow +13″
Time of OT Goals	0′ to 15′; 16′ to 30′; 31′ to 45′*; 45 to 60′; 61 to 75′; 76 to 90′**
Score at the moment	Tied: Balanced; Unbalanced

(i) “Time of the OT Goals”: the time during the game the transition occur; (ii) “Duration of transition”: the time spent on the transitions (iii) Score at the moment: the result verified at the moment of the goal; *: Interval extends to the end of the first half; **: Interval extends until the end of the game.

Five researchers underwent initial training sessions spanning 8 h over 3 days to observe and analyze specific events. Subsequently, they independently conducted observations and codifications using a standardized instrument. Subsequently, the researchers were provided with 30 events to analyze and code independently. After three weeks from the initial training, the analysis of events was revisited, with only those researchers demonstrating homogeneous observation and analysis criteria among themselves continuing in the study. This refinement left three researchers. This iterative process was repeated a second time until consensus was reached among all observers and analysts.

### Statistical analysis

The assumptions of normality and heterogeneity were verified with the Kolmogorov-Smirnov and Levene tests, respectively. Following this verification multiple Multinomial Logistic Regression analyses investigate the association between the dependent variable “Duration of the transition” and the independent variables (see Table 1). To assess multicollinearity, Variance Inflation Factor (VIF) values were calculated, ensuring they remained below the threshold of 10, and collinearity tolerance was monitored. The independence of residuals was evaluated using the Durbin-Watson statistics. A stepwise regression method was employed to identify categories and predictors influencing the likelihood of scoring goals through Offensive Transitions, enhancing the understanding of the impact that these transitions have on the dynamics of a game within each group of leagues. All the analysis were performed by Jamovi—Software.

### Ethics approval and consent to participate

All the video footage used is publicly available. Thus no informed consent or ethics committee approval was required (18, 19). Also, once the analyzed data was retrospective, the athletes performed during their competitive season, and images of the games were broadcast on free-to-air TV. Ethics Methods Committee clearance was not required (18). By informing all participating players, all tracking complies with the general data protection regulation (GDPR) https://gdpr-info.eu/, accessed 07/20/20. Nevertheless, the research received approval from the Ethics Committee of the University of Maia (37/2021) and was conducted in compliance with the guidelines of the Declaration of Helsinki.

## Results

### Descriptive analysis

Of the 3,497 goals recorded across 1,151 matches, 1,649 were produced through direct offensive transitions, representing 47% of the total goals scored. This impact is even more pronounced in the top leagues, with 53% of goals being scored through offensive transitions, followed by Marginal leagues at 43% and Emerging leagues at 42%. The outcomes achieved in each of these leagues are detailed in Supplementary Table S1. Twenty to 23% (21% Top Leagues, 20% Marginal Leagues and 23% Emerging Leagues) of goals scored through offensive transitions stem from positive outcomes.

Regarding the timing of goals through offensive transition, all league groups show the highest percentages in the 76–90** interval, meaning that over 25% of the goals obtained through transition occur during this period. Combining these values with the period leading up to halftime (31′–45′), the results show that 45% of offensive transition goals are scored during these periods in the Top Leagues, 44% in the Marginal Leagues, and 48% in the Emerging Leagues. Examining the transitions observed in instances where the outcome is categorized as “Tied” and “Balanced” (Table 2), the Top Leagues exhibit 80% of offensive transition goals during such scenarios. In comparison, Marginal Leagues showcase 77%, while emerging leagues demonstrate an impressive 84%. Concerning to the time spent in executing offensive transitions, all league groups demonstrate higher percentages in the “Slow +13” category, with percentages ranging between 39% (Top Leagues), 42% (Marginal Leagues) and 37% (Emerging Leagues). Taking into account successful transitions of category “Medium: 8 to 12” alongside these values, the percentages notably rise to 69% in the Top Leagues, 70% in the Marginal Leagues, and 68% in the Emerging Leagues. This indicates that only one-third of successful transitions occur until 8 s inclusive. Table 3 presents the breakdown of transition duration, depicting the time taken for successful transitions, across different game intervals within each league group. Specific league-level outcomes are detailed in Supplementary Table S2.

**Table 2:** Distribution of the offensive transition goals from the studied variables across the three groups classified and relative frequencies.

Performance indicators	Top Leagues % (*n* = 810)	Marginal leagues% (*n* = 494)	Emerging leagues% (*n* = 345)
Variable dependent—duration of transition			
“Fast” 0″ to 8″	**31,10** (*n* = 252)	**30,0** (*n* = 148)	**31,88** (*n* = 110)
“Medium” 9″ to 12″	**29,50** (*n* = 239)	**28,1** (*n* = 139)	**31,30** (*n* = 108)
“Slow” +13″	**39,40** (*n* = 319)	**41,9** (*n* = 207)	**36,52** (*n* = 126)
Variable independent—time of the OT goals			
0′–15′	**12,22** (*n* = 99)	**10,93** (*n* = 54)	**13,33** (*n* = 46)
16′–30′	**12,84** (*n* = 104)	**14,17** (*n* = 70)	**11,01** (*n* = 38)
31′–45′*	**19,38** (*n* = 157)	**19,03** (*n* = 94)	**20,29** (*n* = 70)
45′–60′	**14,32** (*n* = 116)	**13,77** (*n* = 68)	**8,99** (*n* = 31)
61′–75′	**16,05** (*n* = 130)	**17,41** (*n* = 86)	**18,55** (*n* = 64)
76′–90′**	**25,19** (*n* = 204)	**24,70** (*n* = 122)	**27,83** (*n* = 96)
Variable Independent—Score at the moment			
Tied	**32,47** (*n* = 263)	**33,20** (*n* = 164)	**29,57** (*n* = 102)
Balanced	**47,04** (*n* = 381)	**44,33** (*n* = 219)	**53,62** (*n* = 185)
Unbalanced	**20,49** (*n* = 166)	**22,47** (*n* = 111)	**16,81** (*n* = 58)

“Time of the OT Goals”—Goals obtained by Offensive transition. *: Interval extends to the end of the first half; **: Interval extends until the end of the game; Tied: Goal obtained with the result 0–0; Balanced: Goal is obtained with the result until one goal of difference.

Bold values indicate the percentage.

**Table 3:** Distribution of offensive transition goals, per interval of the game and duration of the transition according to the group leagues.

Time of the OT goals	Leagues	Transition duration		
		Fast 0″–8″	Medium 9″–12″	Slow +13″
0′–15′	Top Leagues	28	33	38
	Marginal Leagues	17	17	20
	Emerging Leagues	22	11	13
16′–30′	Top Leagues	35	29	40
	Marginal Leagues	18	22	30
	Emerging Leagues	9	14	15
31′–45′*	Top Leagues	56	40	61
	Marginal Leagues	29	22	43
	Emerging Leagues	24	22	24
45′–60′	Top Leagues	39	34	43
	Marginal Leagues	21	15	32
	Emerging Leagues	14	6	11
61′–75′	Top Leagues	42	37	51
	Marginal Leagues	25	28	33
	Emerging Leagues	18	20	26
76′–90′**	Top Leagues	51	66	87
	Marginal Leagues	38	35	49
	Emerging Leagues	24	35	37
Totals		510	486	653

“Time of the OT Goals”: Goals obtained by Offensive transition. *: Interval extends until the end of the first part; **: Interval extends until the end of the game; Fast 0”–8”: Offensive Transitions completed until seconds; Medium 9”–12”: Offensive Transitions completed between 9 and 12 s; Slow +13”: Offensive Transitions completed in 13 or above seconds.

From the obtained through offensive transition this investigation isolated the goals scored when the result was “balanced”, up to a goal of difference, and when the game was 0–0 (“tied”). This was done to improve the comprehension of the impact of the offensive transitions on a game's dynamics (Table 4).

**Table 4:** Distribution of OT goals, per interval of the game and duration of the transition according with the group leagues.

Time of the OT goals	Leagues	Tied			Balanced		
		Transition duration			Transition duration		
		Fast 0″–8″	Medium 9″–12″	Slow +13″	Fast 0″–8″	Medium 9″–12″	Slow +13″
0′–15′	Top Leagues	2	1	4	26	31	34
	Marginal Leagues	1	3	3	15	14	17
	Emerging Leagues	4	0	2	18	11	11
16′–30′	Top Leagues	9	9	16	21	17	23
	Marginal Leagues	8	6	9	9	16	19
	Emerging Leagues	5	5	10	4	9	5
31′–45′*	Top Leagues	28	21	31	20	14	18
	Marginal Leagues	16	11	16	9	7	19
	Emerging Leagues	10	13	15	8	7	7
45′–60′	Top Leagues	26	18	22	9	8	11
	Marginal Leagues	9	7	15	8	3	9
	Emerging Leagues	7	2	6	3	2	3
61′–75′	Top Leagues	23	18	27	7	6	5
	Marginal Leagues	17	14	18	1	3	3
	Emerging Leagues	12	11	17	1	5	4
76′–90′**	Top Leagues	36	42	48	4	3	6
	Marginal Leagues	23	21	22	2	3	7
	Emerging Leagues	15	26	25	1	1	2
Totals		251	228	434	166	160	203

“Time of the OT Goals”: Goals obtained by Offensive transition. Tied: Goal obtained with the result 0–0; Balanced: Goal is obtained with the result until one goal of difference; *: Interval extends until the end of the first part; **: Interval extends until the end of the game; Fast 0″–8″: Offensive Transitions completed until 8 s; Medium 9″–12″: Offensive Transitions completed between 9 and 12 s; Slow +13″: Offensive Transitions completed in 13 or above seconds.

In the study, multiple multinomial logistic regressions were employed to investigate the association between various variables, specifically “Duration of Transitions”, “Time of the OT Goals”, and “Score at the moment”, and the attainment of goals through direct offensive transitions in different league groups. The results for the Top Leagues (Table 5) demonstrated associations between the variables “Duration of Transitions” and “Time of the OT Goals”, as well as with the variable “Score at the moment”. Thus, the successful offensive transitions from ‘Medium: 9″–12″ when compared to ‘Fast: 0″-8″, have an odds ratio of 1.81, indicating 1.81 times greater odds of occurrence when they happen in the last 15 min of the game compared to 15 min before halftime [95% CI (1.05–3.13)]. Table 5 also demonstrated that the slower successful offensive transitions, when compared to the faster ones, have a 1.68 times greater probability of occurring when the game is unbalanced than when it is tied at 0 goals, with a 95% confidence interval of [1.04–2.72]. The odds ratio of 1.68 suggests a positive association between a slower transition and an unbalanced score compared to a tied score. Finally, the multinomial logistic regression data of the Top Leagues showed that the slower successful offensive transitions, when compared to the faster ones, have an odds ratio of 1.57, indicating 1.57 times greater odds of occurring when the game is balanced (up to 1 goal difference), with a 95% confidence interval of [1.00–2.47].

**Table 5:** Multinomial logistic regression of the variables “duration of the transitions”, “time of the OT goals” and “score at the moment” in top leagues.

Duration of the transitions vs. time of the OT goals vs. Score at the moment—top leagues						
Multinomial logistic regression						
Performance indicators		*p*	OR (95% CI)	AIC	BIC	*R* ^2^_McF_
Duration of the Transitions	Time of the OT Goals			1,783	1,839	0.00408
Medium 9″ to 12″—Fast 0″ to 8″	76′–90′**—31′–45′*	0.033	1.81 (1.05–3.13)			
Duration of the Transitions	Score at the moment			1,773	1,801	0.00307
Slow +13″—Fast 0″ to 8″	Unbalanced—Tied	0.034	1.68 (1.04–2.72)			
Slow +13″—Fast 0″ to 8″	Unbalanced—Balanced	0.05	1.57 (1.00–2.47)			

“Time of the OT Goals”: Goals obtained by Offensive transition. Tied: Goal obtained with the result 0–0; Balanced: Goal is obtained with the result until one goal of difference; *: Interval extends until the end of the first part; **: Interval extends until the end of the game; Fast 0″–8″: Offensive Transitions completed until seconds; Medium 9″–12″: Offensive Transitions completed between 9 and 12 s; Slow +13″: Offensive Transitions completed in 13 or above seconds. *p*, *p* value; OR, odd ratios; 95% CI, confidence intervals (95%); AIC, akaike information criterion; BIC: bayesian information criterion; *R*^2^_McF_, McFadden's *R*^2^.

Despite executing the identical Multinomial Logistic Regression model in the Marginal Leagues, no relevant associations were found among the variables “Duration of Transitions”, “Time of the OT Goals”, and “Score at the moment”. It was observed that no association existed between the “Duration of Transitions”, the “Time of the OT Goals”, and the “Score at the moment” to obtain goals from successful Offensive Transitions.

Regarding the Emerging Leagues the associations of the Multinomial Logistic Regression model founded are presented in Table 6.

**Table 6:** Multinomial logistic regression of the variables “duration of the transitions”, “time of the OT goals” in emerging leagues.

Duration of the transitions vs. time of the OT goals—emerging leagues						
Multinomial logistic regression						
Performance indicators		*p*	OR (95% CI)	AIC	BIC	*R* ^2^_McF_
Duration of the Transitions	Time of the OT Goals			768	814	0.0168
Medium 9″ to 12″—Fast 0″ to 8″	16′–30′—0′–15′	0.044	3.11 (1.03–9.41)			
Medium 9″ to 12″—Fast 0″ to 8″	76′–90′**—0′–15′	0.019	2.92 (1.20–7.11)			
Slow +13″—Fast 0″ to 8″	76′–90′**—0′–15′	0.028	2.61 (1.11–6.15)			
Medium 9″ to 12″—Fast 0″ to 8″	16′–30′—45′–60′	0.047	3.63 (1.02–12.94)			
Medium 9″ to 12″—Fast 0″ to 8″	76′–90′**—45′–60′	0.027	3.40 (1.15–10.10)			

“Time of the OT Goals”: Goals obtained by Offensive transition; **: Interval extends until the end of the game; Fast 0″–8″: Offensive Transitions completed until seconds; Medium 9″–12″: Offensive Transitions completed between 9 and 12 s; Slow +13″: Offensive Transitions completed in 13 or above seconds. *p*, *p* value; OR, odd ratios; 95% CI, confidence intervals (95%); AIC, akaike information criterion; BIC, bayesian information criterion; *R*^2^_McF_, McFadden's *R*^2^.

On Emerging leagues, the successful offensive transitions that take a medium amount of time, when compared to fast ones, have a likelihood of 3,11 times greater odds of occurrence when they happen in the second 15 min of the game compared to the initial 15 min [95% CI (1.03–9.41)]. The offensive transitions that result in a goal directly or by a positive outcome, that take a medium amount of time, when compared to fast ones, have an odds ratio of 2.92, indicating 2.92 times greater odds of occurrence when they happen in the last 15 min of the game compared to the first 15 min [95% CI (1.20–7.11)]. Similarly, the successful offensive transitions that take a slower time, when compared to fast ones, have an odds ratio of 2.61, indicating 2.61 times greater odds of occurrence when they happen in the last 15 min of the game compared to the first 15 min [95% CI (1.11–6.15)]. Finally, the successful offensive transitions that take a medium amount of time, when compared to fast ones, have an odds ratio of 3.63, indicating 3.63 times greater odds of occurrence when they happen from 15′ to 30′ compared to the first 15 min of the second half of the game [95% CI (1.02–12.94)]. Similarly, the successful offensive transitions that take a medium amount of time, when compared to fast ones, have an odds ratio of 4.40, indicating 4.40 times greater odds of occurrence when they happen from 75′ to 90′ compared to the first 15 min of the second half of the game [95% CI (1.15–10.10)].

In each league group, a Multinomial Logistic Regression model was employed, focusing exclusively on successful offensive transitions leading to a goal when the match was tied (0–0) and when the game was deemed “balanced” (with up to a one-goal difference), analyzed separately. In this scenario, no associations found between the different Top and Marginal Leagues variables. Notably, the Emerging Leagues were the sole leagues that revealed an association between the variables, as indicated in Table 7.

**Table 7:** Multinomial logistic regression of the variables “duration of the transitions” and “time of the OT goals” considering only the goals while “balanced” in emerging leagues.

Duration of the transitions vs. time of the OT goals—emerging leagues						
Multinomial logistic regression						
Performance indicators		*p*	OR (95% CI)	AIC	BIC	*R* ^2^_McF_
Duration of the Transitions	Time of the OT Goals			413	452	0.0321
Medium 9″ to 12″– Fast 0″ to 8″	76′–90′**—45′–60′	0.037	6.07 (1.12–33.15)			

“Time of the OT Goals”: Goals obtained by Offensive transition and Positive Outcome. **: Interval extends until the end of the game; Fast 0″–8″: Offensive Transitions completed until seconds; Medium 9″–12″: Offensive Transitions completed between 9 and 12 s; *p*, *p* value; OR, odd ratios; 95% CI, confidence intervals (95%); AIC, akaike information criterion; BIC, bayesian information criterion; *R*^2^_McF_, McFadden's *R*^2^.

## Discussion

The objective of the current investigation was to analyze the time spent executing a successful transition across various league groups. The aim was to furnish insights into the temporal aspects of offensive transitions, elucidating when they occur during playing time, the corresponding duration of these transitions, and their impact on the final score. The variables under scrutiny encompassed the transition duration, the timing of the offensive transition within the game, and the score when the goal was achieved.

The study provided a comprehensive understanding of how goals scored through direct offensive transitions and positive outcomes impact the game's unfolding. Across all league groups, it is observable that these occurrences are more prevalent in the last 15 min of each half, with particular emphasis on the final 15 min of the game. In top leagues, these two intervals account for 44% of goals obtained through offensive transitions, while in Marginal leagues, is 45%, and in Emerging leagues, it is 47%. This pattern signifies a consistent trend in goal acquisition and event similarity regardless of the analyzed league group. These numbers highlight that the last 15 min of each half are critical for goal attainment through offensive transitions. These timeframes, which precede halftime and the end of the game, are characterized by a high cumulative physical strain.

Several studies have provided evidence that both elite and sub-elite football players' ability to perform high-intensity exercise is reduced toward the end of games (20–22). Also, Paquettte and collaborators (23), in their research have shown the cumulative effect of stress factors, that can physical (cumulative workload), and non-physical (emotional or psychological), can decrease an individual's load capacity, which may be reflected in their response to external demands. This phenomenon explains the challenges associated with defensive recovery after a loss of possession, and the re-establishment of defensive positioning. The exchange from the offensive phase to the defensive phase requires an adaptation period due to the differentiated behaviors that must be adjusted (24). This adjustment period gives rise to imbalances that favor the team engaged in offensive transition, creating advantages for them.

## Top leagues

Considering the associations between identified variables in the Top Leagues, it is safe to assert that medium-speed transitions gain relevance in the game's final quarter compared to the last quarter of the first half. This occurrence indicates that, beyond the physical aspect, teams take more risks in pursuit of scoring, leading to positional imbalance. In their defensive processes to maintain a lead, teams tend to lower their defensive lines. As a result, upon ball recovery, they initiate transitions further away from the opposing goal, thereby increasing the execution time of a successful transition, which goes in the same line as the research of Eusebio and collaborators (25), that demonstrated that a relevant number of successful offensive transitions starts in the defensive midfield. Consequently, transitions during this timeframe are prone to medium and long duration. These data diverge from the findings of various studies and authors suggesting that the effectiveness of offensive success increases the closer the transition is to the opponent's goal (4, 26). It is also important to mention that fast transitions are important during the game, namely in the other periods.

The identified associations regarding the match score during goal acquisition and the time spent in offensive transitions also reveal that teams losing by more than one goal concede more goals due to slow offensive transitions (+13″) than when the game is balanced or tied. This underscores the risk-taking behavior of teams at a disadvantage, striving to score and re-enter the match. Similarly, teams losing by a single goal are more susceptible to conceding goals through offensive and prolonged transitions, confirming that teams take risks and become disorganized, while those in the lead defend closer to their goal. These results are consistent with findings from various studies that have shown that when teams are winning, they use a low-pressure defense line (close to their own goal) to maintain the scoreline and to explore imbalances from the attacking team and their “advanced position” when they lose possession (27–29).

## Marginal leagues

Regarding Marginal Leagues, goals through offensive transitions exhibit a lower success rate in the early periods of each half, suggesting a higher balance in positioning and an initial strategy of teams with greater expectations and, consequently, lower exposure to risk. These leagues are characterized by a relevant percentage of successful slow transitions (42%), indicating that these transitions initiate further from the opponent's goal with more elaboration. In future studies, it would be interesting to analyze the number of participants in this league group and others to understand if the duration of offensive transitions is related to the number of participants. Including the Russian league in this group is noteworthy, considering that a substantial part of the championship is played under challenging weather conditions, with snow being a frequent presence. According to Gatterer and collaborators (30), the effect of cold in performance, in variables as time to fatigue, finish time or velocity “is clear”. The same authors mention that even with the process of acclimatization that players undergo, their performance is affected, although there are strategies attempting to mitigate this, notably through exclusive warm-up protocols and the use of insulative clothing technology. The particularity of football lies in the constant exposure to outdoor conditions and the playing field itself (whether through tackles and goalkeeper dives), interactions with the ball, its contact with the field, and players. These factors should be considered in future research endeavors. Illmer and Daumann (31), state clearly in their study that snowfall has a significant negative effect on technical performances regarding the duels that players play during the game. This is supported by the fact that transitions lasting more than 9 s account for 70% of all successful offensive transitions. It is also intriguing to note that “fast” transitions predominantly occur in the second halves of games, suggesting that teams strategically alter their game approaches (Table 1), namely changing the defensive pressure zones to more advanced areas of the field of play, which leads to an increase in possession recoveries in these areas (32) and consequently shorter attacks. Additionally, Table 3 reveals that Marginal Leagues have a significantly higher number of successful transitions in the first half when the game has a goal difference of up to one goal (excluding a 0–0 result). Conversely, transitions preferentially occur in the second half when the score is tied (0–0). These results demonstrate that teams, as time progresses and the game approaches its conclusion, tend to expose themselves more to imbalances that a loss of possession may cause, particularly when the score is 0–0. Performances are impacted more significantly in the physical aspect rather than the technical aspect (31) indicating that as the game progresses and fatigue and accumulated stress set in, players are more prone to higher strategic risk-taking.

## Emerging leagues

Regarding Emerging Leagues, analyzing Table 1 reveals that successful transitions occur in more than half of the instances (52%) when the score is “balanced”, emphasizing the significance of successful transitions during the scoreline. This value should be associated with the fact that the last quarter of the game is responsible for 28% of the total successful offensive transitions, clearly indicating their considerable impact as the games approach their conclusion. Multiple factors explain the high number of associations between transition duration variables and the game periods in which successful transitions occur. Although various authors have demonstrated that fast transitions lead to a higher shot conversion rate (7, 8), it does not necessarily indicate that these shooting opportunities have been successful in a greater proportion than medium and long-duration transitions.

The lower competitive experience of many players on the rosters and the weather conditions that significantly impact the athletes' physical condition partly explain team behaviors and the difficulty in defensive recovery in the final quarters preceding the end of the first half and the game. Different authors in their research (31, 33), have found that although playing in significantly high temperatures does not notably affect the performance of technical aspects, (and in fact, the quality and quantity of technical gestures may increase), there are physical constraints, particularly in the distances covered by athletes and the number of sprints they perform per game. The same authors, mention that even though their maximum speeds remain unchanged, playing in conditions of high humidity and temperature influences performances.

Therefore, the strategic approach of teams is notably influenced, especially towards the end of games when internal and external stress factors become more apparent. The tactical immaturity combined with the emotional aspect is a crucial factor, characteristic of the region to which the emerging leagues belong. This factor particularly comes into play towards the end of games when teams strategically reach the “all or nothing” point, which results in obtaining goals but often the failure to score and conceding goals through offensive transitions. This is evident in the fact that emerging leagues were the only ones to demonstrate association between transition duration variables and the period in which these transitions occur, particularly when considering goals in balanced game situations. These associations demonstrate a higher likelihood of successful transitions occurring in the last quarter of the game compared to the last quarter of the first half. The quality of decision-making gains prominence over the speed of the transition in these leagues.

## Significance of analyzing plays preceding set pieces in offensive transitions

Moreover, this article analyzing the plays preceding set pieces proves significant for academic understanding and should be embraced in future studies. By considering positive outcomes arising from offensive transitions as integral to the transitions themselves, the research demonstrates that 20 to 23% of total goals from offensive transitions stem from positive outcomes (25). Previous studies, from Armatas and collaborators (34) Yiannakos & Armatas (8) presented these values as the total goals from offensive transitions alone, but this research also underscores the importance of considering plays preceding set piece goals. More crucial than merely analyzing the goal itself is understanding its origin and the factors enabling its attainment. Thus, a simplistic and narrow analysis of the goal becomes a limitation and outdated, emphasizing the broader significance for coaches and players to examine the genesis of goals.

## Study limitations

The study has limitations that should be acknowledged. Firstly, the analysis focuses solely on the temporal aspects of offensive transitions and does not consider other factors that may influence goal attainment, such as individual player skills or team tactics. Additionally, the study's findings are based on observational data, which may be subject to limitations in data collection methods. Furthermore, the study's sample size and scope are limited to specific league groups, potentially limiting the generalizability of the findings to other contexts. Finally, the study does not explore the impact of external factors such as weather conditions or referee decisions on offensive transitions and goal attainment.

## Conclusion

This investigation examined performance variables to understand how successful offensive transitions influence goal attainment and their impact on the course of the game. The periods in which transitions occurred, the current match score, and the time spent executing offensive transitions were considered. In all league groups analyzed, successful offensive transitions proved an important means of scoring goals. The 15 min preceding the end of the first half and the game's conclusion emerged as decisive periods for scoring goals through offensive transitions. Further analyses revealed that successful offensive transitions in Top Leagues tend to be slower towards the end of the games. The study also indicated that only 31% of successful transitions last up to 8 s. In Marginal Leagues, 42% of successful transitions last 13 s or more, while in Emerging Leagues, the prominence of offensive transitions in balanced games is evident. Overall, the findings underscore the importance of offensive transitions in achieving team objectives. The study emphasizes the prevalence of medium and slow-speed offensive transitions, highlighting the crucial role of decision-making in enhancing goal-scoring opportunities. These results contribute to a deeper understanding of the dynamics and strategies involved in successful offensive transitions, aligning with previous studies indicating that a relevant number of successful transitions originate from deep defensive positions, requiring more time to reach the goal.

## Practical application

The findings of this study offer several practical applications for coaches, analysts, and teams in soccer. Coaches can strategically plan their team's offensive transitions by capitalizing on the critical moments identified in the study, particularly the last 15 min of each half. Tactical adjustments can be made based on the timing and duration of offensive transitions, focusing on improving decision-making over the speed of the transition, especially in Emerging Leagues. Training sessions can be tailored to improve players' decision-making skills during offensive transitions, while fitness and conditioning programs can target the physical demands of these critical periods. Scouts and recruitment staff can use the findings to identify players who excel in executing successful offensive transitions, and teams can use the insights to manage games better and evaluate their performance. Individual players can focus on improving specific aspects of their game related to offensive transitions, such as decision-making, speed, and positioning, contributing to their overall development as soccer players. These practical applications can help teams and individuals enhance their performance in soccer matches by optimizing their offensive transition strategies and decision-making processes. Long ball possessions should be reconsidered and utilized from a positional control perspective rather than solely as a means of goal acquisition.

## Data Availability

The raw data supporting the conclusions of this article will be made available by the authors, without undue reservation.
