# Supplementary material for: Duration, score, and timing: factors influencing the success of offensive transitions in top, marginal, and emerging football leagues
Source: Front Sports Act Living. 2025 Jan 10;6:1462932. doi: 10.3389/fspor.2024.1462932 (PMC11757885; doi:10.3389/fspor.2024.1462932)
Supplement: Supplementary file 1 [file Table1.docx]

Supplementary Information

For

“Duration, Score, and Timing: Factors Influencing the Success of Offensive Transitions in Top, Marginal, and Emerging Football Leagues”

By

Pedro Eusebio*, Pablo Prieto-González, Rui Marcelino

*Corresponding author: eusebio.pedro@gmail.com

Supplementary information with the results obtained in each of the observed leagues is presented below:

Table S1: Distribution of the types of goals per league

|  |  | Games Observed with Goals | Total Goals | Goals by  NT + SP | Goals by  OT + POS OUT | % Of goals By  OT + POS OUT |
| --- | --- | --- | --- | --- | --- | --- |
| Top Leagues | **Germany** | 148 | 492 | 211 | 281 | 57,11 % |
|  | **Italy** | 177 | 540 | 257 | 283 | 52,41 % |
|  | **Spain** | 173 | 494 | 248 | 246 | 49,80 % |
| Totals |  | 498 | 1526 | 716 | 810 |  |
| Marginal Leagues | **Netherland** | 132 | 483 | 275 | 208 | 43,06 % |
|  | **Portugal** | 139 | 368 | 201 | 167 | 45,38 % |
|  | **Russia** | 110 | 293 | 174 | 119 | 40,61 % |
| Totals |  | 381 | 1144 | 650 | 494 |  |
| Emerging Leagues | **UAE** | 86 | 275 | 165 | 110 | 40,00 % |
|  | **Qatar** | 63 | 211 | 116 | 95 | 45,02 % |
|  | **Saudi** | 113 | 341 | 201 | 140 | 41,06 % |
| Totals |  | 262 | 827 | 482 | 345 |  |
| Totals |  | 702 | 3497 | 1848 | 1649 |  |

Note: NT + SP – Goals where there is no offensive transition reported (direct or Positive outcome). Goals from Non-transitions and Set Pieces. OT + POS OUT – Goals obtained by Offensive transition and Positive Outcome.

 Table S2. Distribution of relative frequencies from the studied variables across the nine leagues classified.

|  | Top Leagues % (n=810) | | | Marginal Leagues % (n=494) | | | Emerging Leagues % (n=345) | | | |
| --- | --- | --- | --- | --- | --- | --- | --- | --- | --- | --- |
| Performance indicators | **Germany**  **(n = 281)** | **Italy**  **(n = 283)** | **Spain**  **(n = 246)** | **Netherland**  **(n = 208)** | **Portugal**  **(n = 167)** | **Russia**  **(n = 119)** | **UAE**  **(n = 110)** | **Qatar**  **(n = 95)** | **Saudi**  **(n = 140)** |  |
| Variable Dependent - Duration of Transition | | | | | | | | | |  |
| “Fast”  0’’ to 8’’ | **39,29**  (n= 55) | **24,21**  (n= 23) | **30,00**  (n= 33) | **30,29**  (n= 63) | **36,53**  (n= 61) | **20,17**  (n= 24) | **30,00**  (n= 33) | **24,21**  (n= 23) | **39,29**  (n= 55) |  |
| “Medium”  9’’ to 12’’ | **27,14**  (n= 38) | **44,00**  (n= 38) | **29,09**  (n= 32) | **26,44**  (n= 55) | **25,75**  (n= 43) | **34,45**  (n= 41) | **29,09**  (n= 32) | **40,00**  (n= 38) | **27,14**  (n= 38) |  |
| “Slow”  +13’’ | **33,57**  (n= 47) | **35,79**  (n= 34) | **40,91**  (n= 45) | **43,27**  (n= 90) | **37,72**  (n= 63) | **45,38**  (n= 54) | **40,91**  (n= 45) | **35,79**  (n= 34) | **33,57**  (n= 47) |  |
| Variable Independent - Time of the OT Goals | | | | | | | | | |  |
| 0’ – 15’ | **13,52**  (n= 38) | **9,89**  (n= 28) | **13,41**  (n= 33) | **11,06**  (n= 23) | **10,18**  (n= 17) | **11,76**  (n= 14) | **7,27**  (n= 8) | **16,84**  (n= 16) | **15,71**  (n= 22) |  |
| 16’ – 30’ | **12,10**  (n= 34) | **17,31**  (n= 49) | **8,54**  (n= 21) | **15,87**  (n= 33) | **12,57**  (n= 21) | **13,45**  (n= 16) | **10,91**  (n= 12) | **13,68**  (n= 13) | **9,29**  (n= 13) |  |
| 31’ – 45’* | **20,28**  (n= 57) | **16,96**  (n= 48) | **21,14**  (n= 52) | **18,75**  (n= 39) | **20,36**  (n= 34) | **17,65**  (n= 21) | **21,82**  (n= 24) | **20,00**  (n= 19) | **19,29**  (n= 27) |  |
| 45’ – 60’ | **14,59**  (n= 41) | **16,61**  (n= 47) | **11,38**  (n= 28) | **13,94**  (n= 29) | **14,37**  (n= 24) | **12,61**  (n= 15) | **10,91**  (n= 12) | **5,26**  (n= 5) | **10,00**  (n= 14) |  |
| 61’ – 75’ | **12,81**  (n= 36) | **18,37**  (n= 52) | **17,07**  (n= 42) | **15,38**  (n= 32) | **19,16**  (n= 32) | **18,49**  (n= 22) | **20,00**  (n= 22) | **20,00**  (n= 19) | **16,43**  (n= 23) |  |
| 76’ – 90’** | **26,69**  (n= 75) | **20,85**  (n= 59) | **28,46**  (n= 70) | **25,00**  (n= 52) | **23,35**  (n= 39) | **26,05**  (n= 31) | **29,09**  (n= 32) | **24,21**  (n= 23) | **29,29**  (n= 41) |  |
| Variable Independent - Score at the moment | | | | | | | | | |  |
| Tied | **28,47**  (n= 80) | **34,63**  (n= 98) | **34,55**  (n= 85) | **28,85**  (n= 60) | **37,72**  (n= 63) | **34,45**  (n= 41) | **20,91**  (n= 23) | **33,68**  (n= 32) | **33,57**  (n= 47) |  |
| Balanced | **50,53**  (n= 142) | **44,88**  (n= 127) | **45,53**  (n= 112) | **43,27**  (n= 90) | **43,71**  (n= 73) | **47,06**  (n= 56) | **59,09**  (n= 65) | **52,63**  (n= 50) | **50,00**  (n= 70) |  |
| Unbalanced | **21,00**  (n= 59) | **20,49**  (n= 58) | **19,92**  (n= 49) | **27,88**  (n= 58) | **18,56**  (n= 31) | **18,49**  (n= 22) | **20,00**  (n= 22) | **13,68**  (n= 13) | **16,43**  (n= 23) |  |

 Table S3: Distribution of OT Goals, per interval of the game and duration of the transition according with each league.

| Time of the OT Goals | Leagues |  | Transition Duration | | |  |
| --- | --- | --- | --- | --- | --- | --- |
|  |  |  | **Fast 0’’ – 8’’** | **Medium9’’ – 12’’** | **Slow  +13’’** |  |
| 0’ – 15’ | Top Leagues | Germany | 9 | 12 | 17 |  |
|  |  | Italy | 11 | 9 | 8 |  |
|  |  | Spain | 8 | 12 | 13 |  |
|  | Marginal Leagues | Netherlands | 7 | 5 | 11 |  |
|  |  | Portugal | 4 | 8 | 5 |  |
|  |  | Russia | 6 | 4 | 4 |  |
|  | Emerging Leagues | UAE | 5 | 0 | 3 |  |
|  |  | Qatar | 9 | 3 | 4 |  |
|  |  | Saudi | 8 | 8 | 6 |  |
| 16’ – 30’ | Top Leagues | Germany | 10 | 10 | 14 |  |
|  |  | Italy | 18 | 11 | 20 |  |
|  |  | Spain | 7 | 8 | 6 |  |
|  | Marginal Leagues | Netherlands | 11 | 10 | 12 |  |
|  |  | Portugal | 6 | 7 | 8 |  |
|  |  | Russia | 1 | 5 | 10 |  |
|  | Emerging Leagues | UAE | 4 | 3 | 5 |  |
|  |  | Qatar | 1 | 7 | 5 |  |
|  |  | Saudi | 4 | 4 | 5 |  |
| 31’ – 45’* | Top Leagues | Germany | 20 | 16 | 21 |  |
|  |  | Italy | 18 | 9 | 21 |  |
|  |  | Spain | 18 | 15 | 19 |  |
|  | Marginal Leagues | Netherlands | 13 | 8 | 18 |  |
|  |  | Portugal | 12 | 8 | 14 |  |
|  |  | Russia | 4 | 6 | 11 |  |
|  | Emerging Leagues | UAE | 9 | 7 | 8 |  |
|  |  | Qatar | 4 | 8 | 7 |  |
|  |  | Saudi | 11 | 7 | 9 |  |
| 45’ – 60’ | Top Leagues | Germany | 17 | 12 | 12 |  |
|  |  | Italy | 11 | 14 | 22 |  |
|  |  | Spain | 11 | 8 | 9 |  |
|  | Marginal Leagues | Netherlands | 7 | 7 | 15 |  |
|  |  | Portugal | 12 | 4 | 8 |  |
|  |  | Russia | 2 | 4 | 9 |  |
|  | Emerging Leagues | UAE | 6 | 1 | 5 |  |
|  |  | Qatar | 1 | 3 | 1 |  |
|  |  | Saudi | 7 | 2 | 5 |  |
| 61’ – 75’ | Top Leagues | Germany | 13 | 11 | 12 |  |
|  |  | Italy | 16 | 14 | 22 |  |
|  |  | Spain | 13 | 12 | 17 |  |
|  | Marginal Leagues | Netherlands | 8 | 12 | 12 |  |
|  |  | Portugal | 13 | 8 | 11 |  |
|  |  | Russia | 4 | 8 | 10 |  |
|  | Emerging Leagues | UAE | 5 | 6 | 11 |  |
|  |  | Qatar | 5 | 6 | 8 |  |
|  |  | Saudi | 8 | 8 | 7 |  |
| 76’ – 90’** | Top Leagues | Germany | 15 | 31 | 29 |  |
|  |  | Italy | 15 | 18 | 26 |  |
|  |  | Spain | 21 | 17 | 32 |  |
|  | Marginal Leagues | Netherlands | 17 | 13 | 22 |  |
|  |  | Portugal | 14 | 8 | 17 |  |
|  |  | Russia | 7 | 14 | 10 |  |
|  | Emerging Leagues | UAE | 4 | 15 | 13 |  |
|  |  | Qatar | 3 | 11 | 9 |  |
|  |  | Saudi | 17 | 9 | 15 |  |

Table S4: Distribution of OT Goals, per interval of the game, duration of the transition according and score at the moment in each league

| Time of the OT + POS OUT Goals | Leagues |  | Tied | | | Balanced | | | |
| --- | --- | --- | --- | --- | --- | --- | --- | --- | --- |
|  |  |  | **Transition Duration** | | | **Transition Duration** | | | |
|  |  |  | **Fast 0’’ – 8’’** | **Medium 9’’ – 12’’** | **Slow  +13’’** | | **Fast 0’’ – 8’’** | **Medium 9’’ – 12’’** | **Slow  +13’’** |
| 0’ – 15’ | Top Leagues | Germany | 7 | 11 | 14 | | 2 | 0 | 3 |
|  |  | Italy | 11 | 9 | 8 | | 0 | 0 | 0 |
|  |  | Spain | 8 | 11 | 12 | | 0 | 1 | 1 |
|  | Marginal Leagues | Netherlands | 6 | 3 | 10 | | 1 | 2 | 1 |
|  |  | Portugal | 4 | 7 | 3 | | 0 | 1 | 2 |
|  |  | Russia | 5 | 4 | 4 | | 0 | 0 | 0 |
|  | Emerging Leagues | UAE | **4** | **0** | 3 | | 1 | 0 | 0 |
|  |  | Qatar | 8 | 3 | 3 | | 1 | 0 | 1 |
|  |  | Saudi | 6 | 8 | 5 | | 2 | 0 | 1 |
| 15’ – 30’ | Top Leagues | Germany | 5 | 4 | 10 | | 4 | 4 | 4 |
|  |  | Italy | 13 | 8 | 10 | | 4 | 3 | 10 |
|  |  | Spain | 3 | 5 | 3 | | 1 | 2 | 2 |
|  | Marginal Leagues | Netherlands | 5 | 7 | 7 | | 5 | 3 | 4 |
|  |  | Portugal | 3 | 5 | 4 | | 3 | 2 | 3 |
|  |  | Russia | 1 | 4 | 8 | | 0 | 1 | 2 |
|  | Emerging Leagues | UAE | 2 | 1 | 2 | | 2 | 2 | 3 |
|  |  | Qatar | 0 | 5 | 1 | | 1 | 2 | 4 |
|  |  | Saudi | 2 | 3 | 2 | | 2 | 1 | 3 |
| 30’ – 45’* | Top Leagues | Germany | 6 | 3 | 6 | | 11 | 10 | 12 |
|  |  | Italy | 8 | 4 | 5 | | 7 | 5 | 10 |
|  |  | Spain | 6 | 7 | 7 | | 10 | 6 | 9 |
|  | Marginal Leagues | Netherlands | 3 | 2 | 7 | | 7 | 4 | 7 |
|  |  | Portugal | 5 | 3 | 8 | | 7 | 3 | 3 |
|  |  | Russia | 1 | 2 | 4 | | 2 | 4 | 6 |
|  | Emerging Leagues | UAE | 4 | 2 | 1 | | 2 | 5 | 6 |
|  |  | Qatar | 1 | 2 | 2 | | 2 | 5 | 5 |
|  |  | Saudi | 3 | 3 | 4 | | 6 | 3 | 4 |
| 45’ – 60’ | Top Leagues | Germany | 2 | 2 | 4 | | 13 | 7 | 6 |
|  |  | Italy | 5 | 3 | 3 | | 5 | 8 | 11 |
|  |  | Spain | 2 | 3 | 4 | | 8 | 3 | 5 |
|  | Marginal Leagues | Netherlands | 1 | 1 | 3 | | 4 | 2 | 6 |
|  |  | Portugal | 7 | 1 | 3 | | 3 | 2 | 5 |
|  |  | Russia | 0 | 1 | 3 | | 2 | 3 | 4 |
|  | Emerging Leagues | UAE | 1 | 0 | 1 | | 4 | 1 | 3 |
|  |  | Qatar | 0 | 1 | 1 | | 0 | 1 | 0 |
|  |  | Saudi | 2 | 1 | 1 | | 3 | 0 | 3 |
| 60’ – 75’ | Top Leagues | Germany | 1 | 1 | 1 | | 8 | 7 | 5 |
|  |  | Italy | 2 | 1 | 2 | | 8 | 7 | 14 |
|  |  | Spain | 4 | 4 | 2 | | 7 | 4 | 8 |
|  | Marginal Leagues | Netherlands | 0 | 2 | 1 | | 6 | 6 | 6 |
|  |  | Portugal | 0 | 1 | 2 | | 8 | 3 | 5 |
|  |  | Russia | 1 | 0 | 0 | | 3 | 5 | 7 |
|  | Emerging Leagues | UAE | 0 | 0 | 2 | | 2 | 4 | 8 |
|  |  | Qatar | 1 | 3 | 1 | | 3 | 3 | 5 |
|  |  | Saudi | 0 | 2 | 1 | | 7 | 4 | 4 |
| 75’ – 90’** | Top Leagues | Germany | 1 | 1 | 1 | | 11 | 20 | 15 |
|  |  | Italy | 3 | 0 | 3 | | 8 | 10 | 17 |
|  |  | Spain | 0 | 2 | 2 | | 17 | 12 | 16 |
|  | Marginal Leagues | Netherlands | 0 | 1 | 1 | | 8 | 8 | 10 |
|  |  | Portugal | 2 | 0 | 5 | | 9 | 7 | 7 |
|  |  | Russia | 0 | 2 | 1 | | 6 | 6 | 5 |
|  | Emerging Leagues | UAE | 0 | 0 | 0 | | 2 | 11 | 9 |
|  |  | Qatar | 0 | 0 | 0 | | 2 | 9 | 6 |
|  |  | Saudi | 1 | 1 | 2 | | 11 | 6 | 10 |

Table S4: Multinomial logistic regression of the variables “Duration of the Transitions”, “Time of the OT Goals” and “Score at the moment” in the Germany league

| Duration of the Transitions vs Time of the OT Goals vs Score at the moment – Germany | | | |
| --- | --- | --- | --- |
| Multinomial logistic regression | | | |
| Performance indicators |  | **p** | **OR**  **(95% CI)** |
| Duration of the Transitions | **Time of the OT Goals** |  |  |
| Medium 9’’ to 12’’ – Fast 0’’ to 8’’ | 76’ – 90’** - 31’ – 45’* | 0.039 | 2.58  (1.05- 6.36) |
| Medium 9’’ to 12’’ – Fast 0’’ to 8’’ | 76’ – 90’** - 45’ – 60’ | 0.029 | 2.93  (1.12- 7.66) |
| Fast 0’’ to 8’’ - Slow +13’’ | 45’ – 60’ - 76’ – 90’** | 0.041 | 2.74  (1.04- 7.20) |

Note: *p*: *p* value; OR: Odd ratios; 95% CI: confidence intervals (95%);

Table S5: Multinomial logistic regression of the variables “Duration of the Transitions”, “Time of the OT Goals” and “Score at the moment” in the Italian league

| Duration of the Transitions vs Time of the OT + POS OUT Goals vs Score at the moment – Italian | | | |
| --- | --- | --- | --- |
| Multinomial logistic regression | | | |
| Performance indicators |  | **p** | **OR**  **(95% CI)** |
| Duration of the Transitions | **Score at the moment** |  |  |
| Slow +13’’ – Fast 0’’ to 8’’ | Balanced - Tied | 0.003 | 2.63  (1.40 – 4.93) |
| Slow +13’’ – Fast 0’’ to 8’’ | Unbalanced – Tied | 0.03 | 2.35  (1.07 – 5.16) |

Note: *p*: *p* value; OR: Odd ratios; 95% CI: confidence intervals (95%);

Table S6: Multinomial logistic regression of the variables “Duration of the Transitions”, “Time of the OT Goals” and “Score at the moment” in the Spanish league

| Duration of the Transitions vs Time of the OT Goals vs Score at the moment – Germany | | | |
| --- | --- | --- | --- |
| Multinomial logistic regression | | | |
| Performance indicators |  | **p** | **OR**  **(95% CI)** |
| Duration of the Transitions | **Score at the moment** |  |  |
| Medium 9’’ to 12’’ – Fast 0’’ to 8’’ | Tied - Balanced | 0.04 | 2.14  (1.04 – 4.37) |

Note: *p*: *p* value; OR: Odd ratios; 95% CI: confidence intervals (95%);

Netherlands league on the Multinomial logistic regression of the analyses of the variables “Duration of the Transitions”, “Time of the OT Goals” and “Score at the moment” does not show any associations.

Table S7: Multinomial logistic regression of the variables “Duration of the Transitions”, “Time of the OT Goals” and “Score at the moment” in the Portuguese league

| Duration of the Transitions vs Time of the OT Goals vs Score at the moment – Germany | | | |
| --- | --- | --- | --- |
| Multinomial logistic regression | | | |
| Performance indicators |  | **p** | **OR**  **(95% CI)** |
| Duration of the Transitions | **Time of the OT Goals** |  |  |
| Medium 9’’ to 12’’ – Fast 0’’ to 8’’ | 0’ – 15’ - 45’ – 60’ | 0.033 | 6.00  (1.15- 31.23) |

Note: *p*: *p* value; OR: Odd ratios; 95% CI: confidence intervals (95%);

Table S8: Multinomial logistic regression of the variables “Duration of the Transitions”, “Time of the OT Goals” and “Score at the moment” in the Russia league

| Duration of the Transitions vs Time of the OT Goals vs Score at the moment – Germany | | | |
| --- | --- | --- | --- |
| Multinomial logistic regression | | | |
| Performance indicators |  | **p** | **OR**  **(95% CI)** |
| Duration of the Transitions | **Time of the OT + POS OUT Goals** |  |  |
| Slow +13’’ – Fast 0’’ to 8’’ | 15’ – 30’ - 0’ – 15’* | 0.028 | 15.00  (1.34- 167.67) |

Note: *p*: *p* value; OR: Odd ratios; 95% CI: confidence intervals (95%);

Table S9: Multinomial logistic regression of the variables “Duration of the Transitions”, “Time of the OT Goals” and “Score at the moment” in the UAE league

| Duration of the Transitions vs Time of the OT Goals vs Score at the moment – UAE | | | |
| --- | --- | --- | --- |
| Multinomial logistic regression | | | |
| Performance indicators |  | **p** | **OR**  **(95% CI)** |
| Duration of the Transitions | **Time of the OT Goals** |  |  |
| Medium 9’’ to 12’’ – Fast 0’’ to 8’’ | 76’ – 90’** - 31’ – 45’* | 0.037 | 4.82  (1.10- 21.19) |
| Medium 9’’ to 12’’ – Fast 0’’ to 8’’ | 76’ – 90’** - 45’ – 60’ | 0.011 | 22.52  (2.07- 245.20) |
| Duration of the Transitions | **Score at the moment** |  |  |
| Medium 9’’ to 12’’ – Fast 0’’ to 8’’ | Balanced - Tied | 0.011 | 6.49  (1.53 – 27.56) |

Note: *p*: *p* value; OR: Odd ratios; 95% CI: confidence intervals (95%);

Table S10: Multinomial logistic regression of the variables “Duration of the Transitions”, “Time of the OT Goals” and “Score at the moment” in the Qatar league

| Duration of the Transitions vs Time of the OT Goals vs Score at the moment – Qatar | | | |
| --- | --- | --- | --- |
| Multinomial logistic regression | | | |
| Performance indicators |  | **p** | **OR**  **(95% CI)** |
| Duration of the Transitions | **Time of the OT Goals** |  |  |
| Medium 9’’ to 12’’ – Fast 0’’ to 8’’ | 15’ – 30’ - 0’ – 15’ | 0.016 | 21.00  (1.78- 248.09) |
| Medium 9’’ to 12’’ – Fast 0’’ to 8’’ | 31’ – 45’** - 0’ – 15’ | 0.048 | 6.00  (1.02- 35.37) |
| Medium 9’’ to 12’’ – Fast 0’’ to 8’’ | 76’ – 90’** - 0’ – 15’ | 0.010 | 11.00  (1.77- 68.35) |
| Slow +13’’ - Fast 0’’ to 8’’ | 76’ – 90’** - 0’ – 15’ | 0.033 | 2,75  (1.16- 39.20) |

Note: *p*: *p* value; OR: Odd ratios; 95% CI: confidence intervals (95%);

Table S11: Multinomial logistic regression of the variables “Duration of the Transitions”, “Time of the OT Goals” and “Score at the moment” in the Saudi league

| Duration of the Transitions vs Time of the OT Goals vs Score at the moment – Saudi | | | |
| --- | --- | --- | --- |
| Multinomial logistic regression | | | |
| Performance indicators |  | **p** | **OR**  **(95% CI)** |
| Duration of the Transitions | **Score at the moment** |  |  |
| Medium 9’’ to 12’’ – Fast 0’’ to 8’’ | Tied - Balanced | 0.029 | 2.85  (1.11 – 7.30) |
| Slow +13’’ – Fast 0’’ to 8’’ | Unbalanced – Tied | 0.03 | 2.35  (1.07 – 5.16) |

Note: *p*: *p* value; OR: Odd ratios; 95% CI: confidence intervals (95%);
